# Complications and Management of a Rare Case of Disseminated Coccidioidomycosis to the Vertebral Spine

**DOI:** 10.1155/2018/8954016

**Published:** 2018-10-08

**Authors:** Sammy G. Nakhla

**Affiliations:** Department of Medicine, Southern Arizona VA Health Care System, Tucson, AZ 85723, USA

## Abstract

Coccidioidomycosis, also known as San Joaquin Valley Fever or Valley Fever, is mostly a pulmonary infection caused by inhalation of spores in an endemic region. Dissemination to bone, joints, meninges, and skin occurs less than one percent of the time. Skeletal involvement accounts for approximately half of the disseminated coccidioidomycosis with the vertebrae as the most common skeletal region. We present a very rare case of disseminated coccidioidomycosis with osteomyelitis and compression fracture of the lumbar vertebral body. This case depicts some of the potential issues that can arise in managing coccidioidomycosis, especially when noncompliance to initial azoles occurs, that can lead to dissemination and complicated bone infections necessitating surgical intervention along with continuous medical therapy.

## 1. Introduction

Coccidioidomycosis is caused by a soil-inhabiting dimorphic spore-forming fungus *Coccidioides immitis* that is endemic in the southwestern region of the United States, Mexico, and parts of South America.

The San Joaquin Valley of California is also a common region for *Coccidioides immitis*, and pulmonary infection can result from inhalation of spores which is commonly referred to as San Joaquin Valley Fever or Valley Fever [1, 2]. The majority of individuals, greater than sixty percent, who are infected are asymptomatic [2]. Disseminated infections to sites such as bones, joints, meninges, and skin account for less than one percent [3]. Skeletal involvement such as osteomyelitis accounts for 20% to 50% of disseminated extrapulmonary infection, with the vertebrae as the most common skeletal site [2].

We report a case of an immunocompetent African American male with disseminated coccidioidomycosis to the vertebral spine. This case illustrates the challenges and potential complications of disseminated coccidioidomycosis of the spine.

## 2. Case Report

A 47-year-old African American male with poorly controlled diabetes mellitus type 1 presented with lower back pain intensifying over several weeks. The patient was born and raised in Ohio but moved away to join the army. While in the military, he was stationed in Germany and Korea. He had moved to Arizona approximately a year prior. The patient had complaints of six months of progressively worse chronic nonproductive cough, night sweats, and significant weight loss. He denied fever, headaches, confusion, gait abnormalities, new joint pain, and skin rashes. Physical examination was grossly unremarkable. The white blood cell (WBC) count was 7,800/*μ*L. Chest X-ray revealed bilateral hilar lymph nodes along with likely granuloma at the right middle lobe. Computed tomography of the chest revealed subcentimeter bilateral pulmonary nodules and middle mediastinal and right hilar lymphadenopathy. He underwent bronchoalveolar lavage with transbronchial lymph node biopsy. The pathology was consistent with granulomatous inflammation and fungal elements consistent with extensive coccidiosis. His cocci serology IDCF qualitative was positive, and the cocci IDCF titer was 8. His HIV test was negative. He was diagnosed with pulmonary coccidioidomycosis and started on oral fluconazole 400 mg daily.

**Figure 1:**
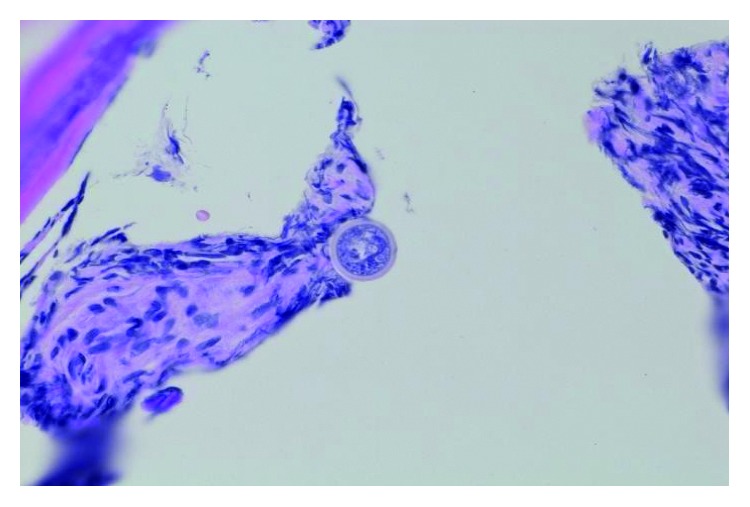
L3 bone biopsy revealing cocci spherules (GMS stain, 400x).

**Figure 2:**
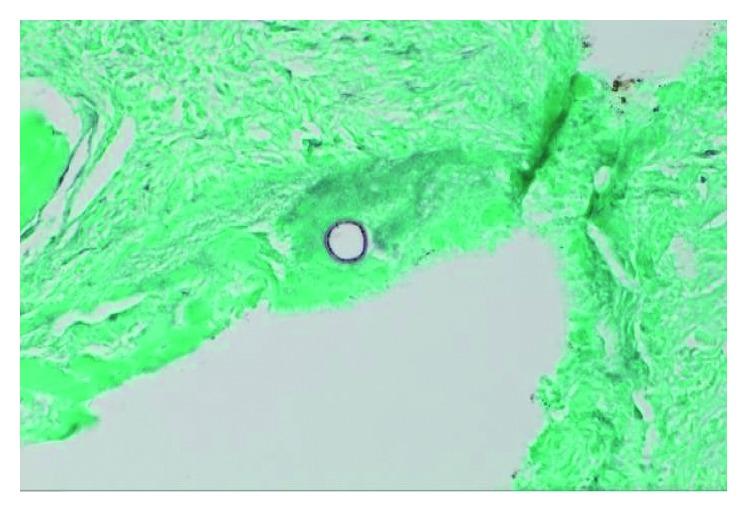
L3 bone biopsy revealing cocci spherules (HE stain, 400x).

**Figure 3:**
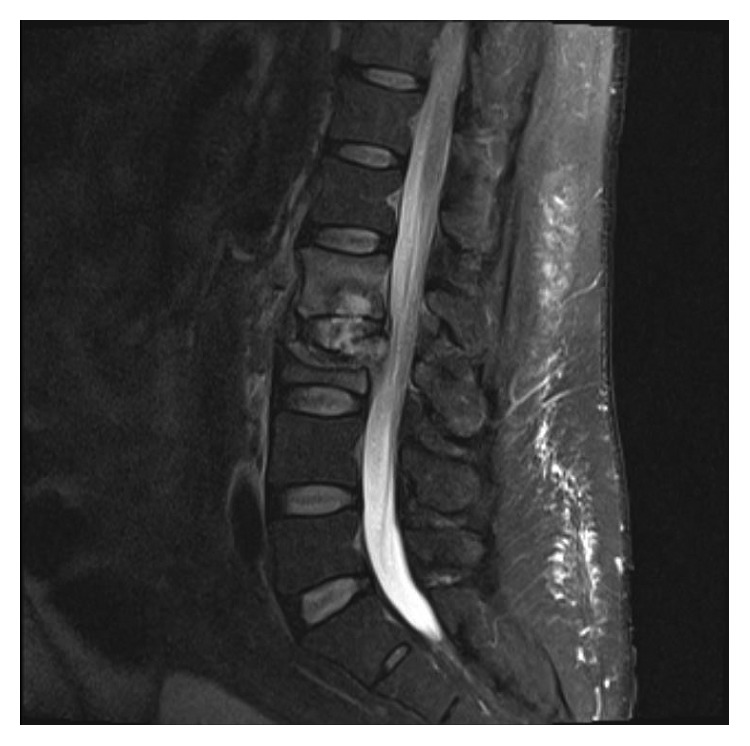
MRI lumbar spine with and without contrast revealing the compression fracture of L3. Ventral epidural enhancement with mass effect on the thecal sac related to an epidural abscess.

The patient had returned to the Emergency Department complaining of worsening lower back pain over the past few weeks. He denied bowel or bladder incontinence and numbness. Physical examination revealed tenderness at the lower back. X-ray of the lumbosacral spine did not show osteomyelitis. Magnetic resonance imaging (MRI) of the lumbar spine without contrast revealed acute-to-subacute superior endplate compression deformity of the L3 vertebral body. Also noted was approximately 10–20% loss of the vertebral body height. No evidence of epidural abscess, spinal canal compromise, neural foraminal narrowing, or spinal cord compression was noted. CT-guided needle biopsy was performed confirming osteomyelitis due to *C. immitis* (Figures 1 and 2). Fluconazole was switched to itraconazole for better bone penetration. Unfortunately, the patient had significant weight gain and lower extremity swelling. Therefore, itraconazole was switched to posaconazole. He subsequently had gradual improvement of lower extremity swelling and weight loss. Subsequent MRI of the lumbar spine with and without contrast revealed T2 hyperintensity within the disc extending into the inferior endplate of L2 and superior endplate of L3. Findings were related to discitis and osteomyelitis. There was also a compression fracture of the superior endplate of L3 with approximately 50% height loss. Finally, there was a mass-like ventral epidural enhancement to the left of the midline with associated mass effect on the thecal sac related to developing an epidural abscess (Figure 3). Neurosurgery was consulted, and the patient underwent L2-L3 laminectomy, L2-L4 posterior spinal fusion, and evacuation of the epidural abscess. The pathology report also confirmed to be positive for *Coccidioides* spherules (Figures 1 and 2). He underwent retreatment with intravenous liposomal amphotericin B (AmBisome) 5 mg/kg of ideal weight for several weeks along with continuing oral posaconazole for life. His repeat cocci IDCF titers decreased from 8 to 2. The patient denied axial pain or radicular pain. His spine remained stable, and he was neurologically intact.

## 3. Discussion

Coccidioidomycosis, also known as Valley Fever, was first described in the region of California's San Joaquin Valley [4]. The coccidioidomycosis spores form in warm wet soils that occur during monsoons or heavy rainfall. The spores are scattered in the air by wind, construction, and farming and become airborne. The lung is the initial area of infection. Most of the infections are asymptomatic and self-limited. Symptoms appear to be flu-like such as fever, cough, headache, chills, night sweats, joint pains, and rashes that typically resolve within a few weeks. Disseminated coccidioidomycosis to sites such as the bone, joints, and skin occurs in less than 1% of individuals. Of those one percent with disseminated coccidioidomycosis, less than half will have vertebral involvement as the most common skeletal site [2]. African Americans, Filipino, Asian, Hispanic descent, impaired T-cell function, chronic steroids use, pregnancy, and antifungal noncompliance are at increased risk for disseminated coccidioidomycosis [4].

Skeletal lesion characteristics are usually well-circumscribed lytic lesions at the vertebral bodies with disc space sparing [5]. Plain radiographs are generally initial evaluations. However, CT and MRI are superior in identifying soft tissue and spinal abnormalities [6]. Other differentials could include metastatic disease, tuberculosis, or other granulomatous diseases. Often, paraspinal soft tissue involvement, abscess, phlegmon, and disk space involvement are seen on imaging [3]. Surgical debridement or stabilization is important and can be critical in preserving neurological stability. Factors that favor surgical intervention are the size of the abscesses, bony sequestration, instability of the spine, or impingement on tissue such as an epidural abscess or spinal cord compression [7]. Medical management along with surgical intervention is often required with aggressive surgical debridement.

Antifungal treatment is the first choice of treatment for coccidioidomycosis. Azoles are recommended for bone and joint coccidioidomycosis. Fluconazole has been the most frequently used antifungal therapy followed by voriconazole and itraconazole [5]. In a comparison of fluconazole 400 mg/day and itraconazole 200 mg bid, the skeletal infections were twice as likely to respond to itraconazole [5]. However, itraconazole should be taken with a full meal, and caution is advised with drug-drug interaction such as proton pump inhibitors and H2 blockers. Posaconazole has been shown to have good bone penetration and may be more effective than itraconazole [9]. Amphotericin B has been recommended for critical bone areas such as the vertebral column and for patients who respond poorly to azoles [5]. This case illustrates several points. First, the importance of early detection and treatment of skeletal involvement should be considered in patients with coccidioidomycosis, especially from endemic areas or immunocompromised ones. Second, the potential issues that can arise in managing coccidioidomycosis, especially when noncompliance to initial azoles occurs, can lead to dissemination and complicated bone infections. Third, for treatment failure to azoles, AmBisome, or both, an alternative tablet posaconazole has been used successfully in cases of disseminated nonmeningeal coccidioidomycosis [10]. Finally, lifelong suppressive therapy is often recommended for these cases [11].
